# Prediction of microbe–drug associations based on a modified graph attention variational autoencoder and random forest

**DOI:** 10.3389/fmicb.2024.1394302

**Published:** 2024-05-31

**Authors:** Bo Wang, Fangjian Ma, Xiaoxin Du, Guangda Zhang, Jingyou Li

**Affiliations:** ^1^College of Computer and Control Engineering, Qiqihar University, Qiqihar, China; ^2^Heilongjiang Key Laboratory of Big Data Network Security Detection and Analysis, Qiqihar University, Qiqihar, China

**Keywords:** microbe–drug association prediction, variational autoencoder, modified graph convolutional neural network, graph attention network, random forest classifier, computational prediction model

## Abstract

**Introduction:**

The identification of microbe–drug associations can greatly facilitate drug research and development. Traditional methods for screening microbe-drug associations are time-consuming, manpower-intensive, and costly to conduct, so computational methods are a good alternative. However, most of them ignore the combination of abundant sequence, structural information, and microbe-drug network topology.

**Methods:**

In this study, we developed a computational framework based on a modified graph attention variational autoencoder (MGAVAEMDA) to infer potential microbedrug associations by combining biological information with the variational autoencoder. In MGAVAEMDA, we first used multiple databases, which include microbial sequences, drug structures, and microbe-drug association databases, to establish two comprehensive feature matrices of microbes and drugs after multiple similarity computations, fusion, smoothing, and thresholding. Then, we employed a combination of variational autoencoder and graph attention to extract low-dimensional feature representations of microbes and drugs. Finally, the lowdimensional feature representation and graphical adjacency matrix were input into the random forest classifier to obtain the microbe–drug association score to identify the potential microbe-drug association. Moreover, in order to correct the model complexity and redundant calculation to improve efficiency, we introduced a modified graph convolutional neural network embedded into the variational autoencoder for computing low dimensional features.

**Results:**

The experiment results demonstrate that the prediction performance of MGAVAEMDA is better than the five state-of-the-art methods. For the major measurements (AUC =0.9357, AUPR =0.9378), the relative improvements of MGAVAEMDA compared to the suboptimal methods are 1.76 and 1.47%, respectively.

**Discussion:**

We conducted case studies on two drugs and found that more than 85% of the predicted associations have been reported in PubMed. The comprehensive experimental results validated the reliability of our models in accurately inferring potential microbe-drug associations.

## 1 Introduction

Microbes, including bacteria, viruses, archaea, fungi, and protists, are dynamic, diverse, and complex gene pools. These microbes form different microbiomes and inhabit different parts of the human body, such as the gut, mouth, vagina, and uterus (Huttenhower et al., 2012). Microbes are considered to be “forgotten” organs that are beneficial to humans, such as assisting the human body in regulating and promoting metabolism by providing protection against pathogens (Gill et al., 2006). In addition, microbes play a crucial role in maintaining the ecological environment within organisms (ElRakaiby et al., 2014). Abnormal growth or decline of microbes can affect human health, likely leading to obesity (de la Cuesta-zuluaga et al., 2023), diabetes (Wen et al., 2008), inflammatory bowel disease (Durack and Lynch, 2019), and even cancer (Schwabe and Jobin, 2013). In recent years, many features of the microbiome and its potential roles in human health have been widely reported. For example, Sprockett et al. (2018) explored how preferential effects affect the microbial community of the gastrointestinal tract in early childhood, and Ximenez and Torres (2017) discussed the development of the microbiome in early life, spanning from pregnancy to birth and extending into the first years of life. In addition, gut microbial communities have been shown to play a key role in cardiometabolic disorders, neuropsychiatric disorders, and cancer. Moreover, some bacteria or viruses can cause very serious infectious diseases, such as COVID-19 (Xiang et al., 2020). Therefore, microbes are considered new therapeutic targets for precision medicine.

Currently, with the rapid increase in drug-resistant pathogenic microbes, it is urgent to determine the association between microbes and drugs to promote subsequent drug development (Ramirez et al., 2016). Recent studies have demonstrated that microbes have an important role in modulating drug activity and toxicity, and drugs can, in turn, influence the diversity and function of microbial communities. There is increasing reporting on the relationship between microbes and drugs. For example, Haiser et al. (2013) noted that the intestinal actinomycete *Eggerthella lenta* is responsible for the inactivation of the cardiac drug digoxin. Yoon et al. (2019) noted that *Enterococcus faecalis* is highly sensitive to imipenem, amikacin, and piperacillin.

Although these microbe–drug associations are obtained from experimental methods, it is practically impossible to identify target microbes, which leads to the slow development of new drugs. In order to overcome this problem, most studies have been devoted to the reuse of known drugs and drug combinations. However, the emergence of drug-resistant microbes poses insightful challenges to drug development. Therefore, there is an urgent need to develop an effective method to infer target microbes with new drug associations. Since traditional wet-lab experiments are time-consuming, labor-intensive, and expensive, computer-based methods can be an effective complement to provide accurate predictions of microbe–drug associations through computation.

At present, existing methods for predicting microbe–drug associations can be classified into three categories: based on network propagation, based on machine learning, and based on deep learning.

(1) Methods based on network propagation

Microbe–drug associations were predicted by constructing a heterogeneous network based on known microbe–drug associations, microbial similarity, and drug similarity. Zhu et al. (2021) designed an HMDAKATZ computational model based on KATZ measurements (Lei and Zhang, 2019) using the chemical structure of the drug to identify potential human microbe–drug associations. The drug was extracted by obtaining the chemical structure of the drug to calculate its similarity with other drugs. The Gaussian interaction profile kernel (van Laarhoven et al., 2011) was then used to calculate the similarity of microbes. Finally, the microbe–microbe similarity network, drug–drug similarity network, and microbe–drug similarity network were combined to construct a microbe–drug similarity network. The potential relationship between microbes and drugs was predicted using the KATZ algorithm. This model can correctly predict microbe–drug association relationships by using a simple metric for heterogeneous networks, but it is not applicable to predict new drugs without known microbial associations or isolated microbes without any known disease associations. Long et al. (2020) used rich biological information to construct a heterogeneous network of microbes and drugs and then utilized a framework based on graph convolutional networks to predict human microbe–drug associations. However, the similarity calculation of microbes (drugs) is still highly dependent on known microbe–drug association information, and the prediction accuracy is not high. Tan et al. (2022) first constructed a heterogeneous network by integrating known microbe–drug associations, microbial similarity, and drug similarity. Then, autoencoder modules based on GAE (Liu et al., 2022) and sparse autoencoder (Jiang et al., 2020) modules are used to learn the topological representation and attribute representation of nodes in the newly constructed heterogeneous network, respectively. Finally, based on these two node representations, two feature matrices for microbes and drugs are constructed separately, and they are used to calculate the possible association scores of microbe–drug pairs. A novel computational model, GSAMDA, based on graph-attentive networks and sparse autoencoder is proposed to infer potential microbe–drug associations, but this model is still not perfect for predicting matrices with sparse data.

(2) Methods based on machine learning

Constructing microbial (drug) profiles uses microbial (drug) similarities and known microbe–disease associations, as well as designing classifiers to identify microbes associated with diseases. Jiang et al. (2020) proposed a computational model based on neighborhood inference and restricted Boltzmann machine (Kirubahari and Amali, 2023). Neighborhood inference can leverage abundant similar information about microbes (drugs), while the restricted Boltzmann machine can learn the latent probability distributions hidden in known microbe–drug associations. Finally, integrated learning is used to combine the individual learners into a stronger predictor. However, this method is not sufficient to reveal the association between drugs and new microbes, or the association between microbes and new diseases, or without any associations. Ma and Liu (2022) integrated multiple sets of data to calculate the functional and semantic similarity of microbes, the structural similarity of drugs, and the information on microbe–drug associations. The hypergraph is constructed using strong neighborhood information. In order to improve the performance of the hypergraph, a simple volume is used to calculate the hyperedge weights. Hypergraph regularization is introduced to the generalized matrix decomposition model, and the higher-order structural information is used to improve the representation of the low-dimensional features. A kind of generalized matrix decomposition based on weighted hypergraph learning (WHGMF) is proposed for predicting potential microbe–drug associations, but using a fixed number of neighbors to construct the hypergraph may limit the adaptability of the model to some extent. In addition, WHGMF uses the generalized matrix decomposition framework, which is not efficient computationally. Yang et al. (2022) proposed a multi-kernel fusion model based on a graph convolutional network, which uses graph convolution to extract multi-layer features, calculates kernel matrices by embedding features on each layer, and fuses multiple kernel matrices based on the average weighting method. Dual Laplacian regularized least squares (Wang et al., 2017) are then used to combine kernels in the microbial and drug spaces to infer new microbe–drug associations.

(3) Methods based on deep learning

The complex heterogeneous network graph of microbe–drug associations was constructed by integrating multi-source bioinformatics data of microbes, drugs, and diseases to extract the non-linear features of microbes and drugs to achieve the prediction of microbe–drug associations. Li et al. (2023) constructed a heterogeneous network of drugs, microbes, and diseases by using multiple sources of biomedical data, then developed a model with a matrix decomposition and a three-layer heterogeneous network to predict potential microbe–drug associations. However, the similarity networks of drugs, microbes, and diseases still have some noise, which leads to this model not being good enough, so there is still more room for improvement. Ma et al. (2023) constructed two heterogeneous microbe–drug networks using multiple similarity metrics of microbes and drugs, as well as known microbe–drug associations or known microbe–disease–drug associations, respectively, and then obtained the feature matrices of microbes and drugs. A computational model, GACNNMDA, based on a graph convolutional neural network was designed to predict the possible scores of microbe–drug pairs. However, this model did not take into account other relevant biological information (e.g., microbial sequences and drug similarity based on side effects), and the prediction accuracy is not high. Long et al. (2020) utilized a variety of sources of biomedical information to construct microbial and multiple networks of drugs. A new integrated framework of graph attention networks with hierarchical attention mechanism and node-level attention was developed for predicting microbe–drug associations from the constructed multiple microbe–drug graphs, but still, there is noise in the features extracted from these similarities. So, this model needs to improve the prediction results.

In order to overcome the inherent defects of the above three types of methods, the investigation set up a new microbe–drug association prediction model named MGAVAEMDA. This model uses a variational autoencoder and incorporates the modified graph convolutional network and graph attention network to improve prediction accuracy in a three-stage process.

The construction process of the MGAVAEMDA model is divided into three steps:

(1) In this part, based on the downloaded microbe–drug associations, the structure information of drugs, and the sequence information of microbes. The microbe–microbe similarity matrix and the drug–drug similarity matrix are obtained through multiple similarity measures and data processing.
(2) In this part, the microbe–microbe similarity matrix, drug–drug similarity matrix, and microbe–drug similarity matrix are inputted into the modified graph variational autoencoder to learn the low-dimensional feature representations of microbes and drugs. The graph attention network is then introduced to extract important features using the attention mechanism to reduce dependence on other biological information.
(3) In this last section, the random forest-based classifier was introduced to calculate the possible scores of microbe–drug associations. Those newly learned important feature representations are combined to form the inputs for the classifier. The final result of the model is voted on by each base classifier.

## 2 Materials and equipment

### 2.1 Microbe–drug associations

In this article, three different datasets, namely MDAD (Sun et al., 2018), aBiofilm (Rajput et al., 2018), and DrugVirus (Andersen et al., 2020), are used to test the predictive power of the MGAVAEMDA model. The MDAD dataset used in the model contains 2,470 associations between 1,373 drugs and 173 microbes. The aBiofilm dataset used in the model includes 2,884 associations between 1,720 drugs and 140 microbes. The DrugVirus database summarizes experimentally confirmed microbe–drug correlations, including 933 associations between 175 drugs and 95 viruses.

Here, an adjacency matrix $\mathrm{MD}\in R^{i\times j}$ was built to preserve the microbe–drug association information. The i represents the number of microbes and j represents the number of drugs. If the microbes $M_{i}$ and drugs $D_{i}$ are related, the entity $MD_{ij}$ has a value of 1, otherwise it is 0.


(1) $$MD_{ij}=\{\begin{array}{lll} 1, & if\;\mathrm{microbe}\;M_{i}\;a\mathrm{ssociated with durg}\;D_{j} & \\ 0, & o\mathrm{therwise} & \end{array}$$

### 2.2 Sequence similarity of microbes

To calculate the sequence similarity of microbial genomes, BLAST (Altschul et al., 1990) can be used to perform a pairwise sequence alignment of microbial genomes. Briefly, the main function of BLAST is to discover regions of local similarity between sequences and then calculate the similarity using a local comparison algorithm (Smith and Waterman, 1981). For example, $M_{A}=m_{A}^{1}m_{A}^{2}\cdots m_{A}^{a}$ and $M_{B}=m_{B}^{1}m_{B}^{2}\cdots m_{B}^{b}$ are asked about the genome sequences of microbe A and microbe B, where a and b are the lengths of sequences $M_{A}$ and $M_{B}$ . BLAST creates the scoring matrix $H_{\left(a+1\right)\times \left(b+1\right)}$ and sets the elements of the first row and column to zero. The element $H_{ij}\left(H_{ij}\in H_{\left(a+1\right)\times \left(b+1\right)}\right),\;\;i=1,2,\ldots \ldots a;j=1,2\ldots b)$ of the formula in this scoring matrix are:


(2) $$\begin{array}{l} H_{ij}=\\ \left\{\begin{array}{c} H_{i-1,j+1}+Score\\ H_{i-k,j}-2\\ H_{i,j-k}-2\\ 0 \end{array}\right.{\left(m_{A}^{i}=m_{B}^{i},Score=1,m_{A}^{i}\neq m_{B}^{i},Score=-1\right)}^{\leftarrow } \end{array}$$

where $m_{A}^{i}$ represents the *i*th sequence value of microbe A, take the matrix $H_{\left(a+1\right)\times \left(b+1\right)}$ , the maximum value is $sw\left(M_{A},M_{B}\right)$ . The similarity between microbe A and microbe B is shown in the following equation:


(3) $$S_{b}\left(A,B\right)=\frac{sw\left(M_{A},M_{B}\right)}{\sqrt{sw\left(M_{A},M_{A}\right)\times sw\left(M_{B},M_{B}\right)}}$$

According to the similarity between two or more microbes, the microbial similarity matrix MSS can be constructed.

### 2.3 Structural similarity of drugs

In this study, SIMCOMP2 was used to search (Hattori et al., 2010) for drug structure similarity. SIMCOMP2 search is a chemical structure search server that provides links to the KEGG PATHWAY database, which contains hand-drawn pathway maps with information on molecular interactions, reactions, and relationships. In the SIMCOMP2 search, by mapping the drugs in the dataset to the drugs in KEGG, the drug structure similarity can be obtained with a cut-off score of 0.01 to filter out drugs with a structure similarity score of 0.01 or higher. Then, matrix DSS is defined to preserve the structural similarity of drugs, where the element $D_{ij}$ represents the drug d(i) and drug $d\left(j\right)$ similarity value.

### 2.4 Gaussian interaction profile kernel similarity of microbes

Gaussian interaction profile kernel similarity (Li et al., 2023) has been widely used in previous studies for the similarity of biological entities. Given the sparse nature of the similarity matrices of microbes and drugs obtained by the above methods, Gaussian interaction profile kernel similarity was constructed based on known microbe–drug associations to obtain a more comprehensive microbial similarity. The matrix MGS represents the microbial Gaussian interaction profile kernel similarity, the matrix element MGS(m(i),m(j)) represents the Gaussian interaction profile kernel similarity of microbes m(i) and m(j) , which is calculated as follows:


(4) $$\mathrm{MGS}\left(m_{\left(i\right)}{\mathrm{,m}}_{\left(j\right)}\right)=\exp\left(-\gamma_{m}∥A_{m\left(i\right)}-A_{m\left(j\right)}{∥}^{2}\right)$$

where $A_{m\left(i\right)}$ represents the *i*th column vector of the adjacency matrix MD as the spectrum kernel for the interactions of microbe m(i) , $\gamma_{m}$ represents the normalized kernel bandwidth of microbe, which can be normalized by the parameter $\gamma_{m}^{\prime }$ . It is calculated as follows:


(5) $$\gamma_{m}=\frac{\gamma_{m}^{\prime }}{\frac{1}{nm}\overset{nm}{\underset{i=1}{\sum }}∥A_{m\left(i\right)}∥\overset{nm}{\underset{i=1}{\sum }}∥A_{m\left(i\right)}{∥}^{2}}$$

### 2.5 Gaussian interaction profile kernel similarity of drugs

Similar to microbes, the Gaussian interaction profile kernel similarity of drugs was calculated. The matrix DGS represents the drug Gaussian interaction profile kernel similarity, and the matrix element $DGS\left(d\left(i\right),\;\;d\left(j\right)\right)$ represents the Gaussian interaction profile kernel similarity of drugs d(i) and d(j) . It is calculated as follows:


(6) $$\mathrm{DGS}\left(d\left(i\right),\;\;d\left(j\right)\right)=\exp\left(-\gamma_{d}∥A_{d\left(i\right)}-A_{d\left(j\right)}{∥}^{2}\right)$$

where $A_{d\left(i\right)}$ represents the *i*th column vector of the adjacency matrix MD as the spectrum kernel for the interactions of drug d(i) , where $\gamma_{d}$ represents the normalized kernel bandwidth of drug.

### 2.6 Similarity fusion

As mentioned above, the similarity of microbes and drugs in different aspects is calculated separately. In order to obtain their comprehensive similarity matrix, the similarities from different perspectives need to be fused. The integrated similarity matrix of microbes is constructed as follows:


(7) $$MS\left(ij\right)=\{\begin{array}{c} \frac{MSS\left(ij\right)+MGS\left(ij\right)}{2},\mathrm{if}\;\;MSS\left(ij\right)\neq 0\\ MGS\left(ij\right),\;\;\mathrm{otherwise} \end{array}$$

The integrated similarity of microbes is the similarity of the Gaussian interaction profile of microbes MGS(ij) if the sequence similarity of microbes MSS(ij)=0, otherwise, it is half of the sum of the two.

Similarly, the integrated similarity matrix of drugs is calculated as follows:


(8) $$DS\left(ij\right)=\{\begin{array}{c} \frac{DSS\left(ij\right)+DGS\left(ij\right)}{2},\mathrm{if}\;\;DSS\left(ij\right)\neq 0\\ DGS\left(ij\right),\;\;\mathrm{otherwise} \end{array}$$

### 2.7 Data processing

In order to reduce noise or fluctuations in the data and make it easier to subsequently analyze the data trends, the combined similarity matrices of microbes and drugs obtained above were smoothed, and for microbes and drugs, the smoothing matrices were calculated using the following formula:


(9) $$SMA=\left(X_{1}+X_{2}+.\ldots +X_{n}\right)/n$$

where $X_{1}$ to $X_{n}$ are the data points within the window, usually from the past n time points. n is the size of the window, which determines the number of data points to be computed within the window. $X_{1}$ to $X_{n}$ correspond to the data within the microbial similarity matrix. Similarly, MS(ij) to MS(i(j+n)) , the same for drugs. Subsequently, several experiments were conducted for the choice of window, and the optimal window value was obtained. By summing the data points within the window and dividing by the window size *n*, a simple moving average at time point j can be calculated. This smoothing technique helps to observe trends, reducing noise and sudden fluctuations for a better understanding of the long-term trend of the data.

The composite matrix was obtained after the curve of the microbe and drug was smoothed. The data were binarized to reduce its complexity, where continuous-type data were transformed into a process containing only two values of data (0 and 1). In binarization, a threshold value is selected, and then each value in the data is compared with that threshold value. If the data are equal to or greater than the threshold, it is mapped to 1. If the data are less than the threshold, it is mapped to 0. In this way, the data are converted into binary form, which is analyzed in the next step. In this study, different thresholds are selected for microbes and drugs, *m*th represents the microbe threshold, and *d*th represents the drug threshold. The binarization matrix of microbes is calculated as follows:


(10) $$MS\left(ij\right)=\left\{\begin{array}{c} 1,\;\;\mathrm{if}\;MS\left(ij\right)\geq mth\\ 0,\mathrm{otherwise} \end{array}\right.$$

The binarized matrix for the same drug is calculated as follows:


(11) $$DS\left(ij\right)=\left\{\begin{array}{c} 1,\;\;\mathrm{if}\;DS\left(ij\right)\geq dth\\ 0,\mathrm{otherwise} \end{array}\right.$$

Finally, the binarized similarity matrix is transformed into an adjacency matrix and feature matrix, where the adjacency matrix represents the connectivity of the graph and the feature matrix represents the features of each node. In the next step, both the adjacency matrix and feature matrix are put into MGAVAEMDA for low-dimensional feature extraction.

## 3 Methods

### 3.1 MGAVAEMDA framework

**Figure 1:**
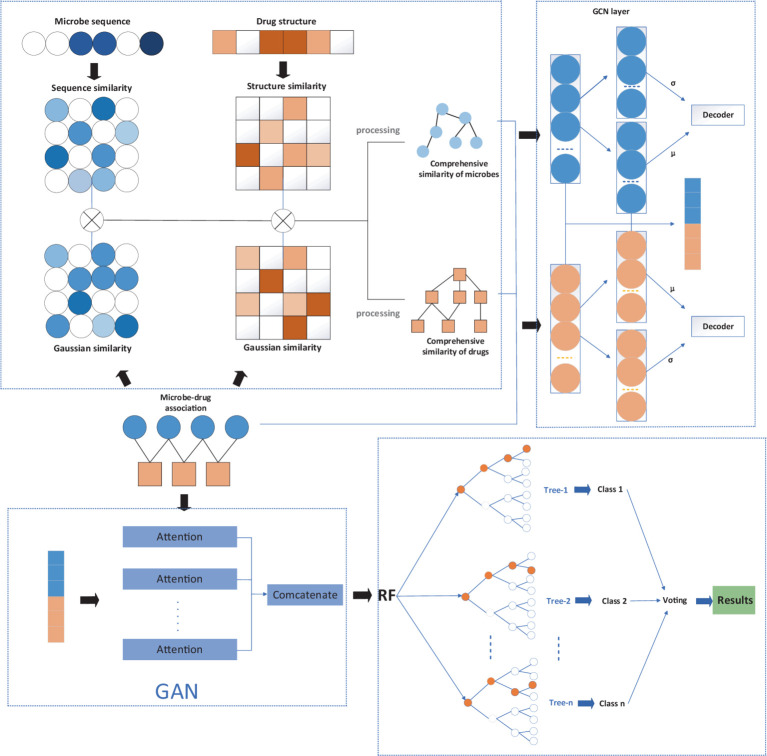
The flowchart of the MGAVAEMDA model.

The flowchart of the MGAVAEMDA model is shown in Figure 1, based on a graph-attentive variational autoencoder. The model is divided into three main steps: (1) construct the similarity networks of microbes and drugs, respectively; (2) extract the feature representations of microbes and drugs using the modified graph attention variational autoencoder (MGAVAE); and (3) embed the combination of microbe and drug representations into the random forest classifier to obtain the final prediction scores.

### 3.2 Variational autoencoder

MGAVAE can reconstruct the node attributes and graph structure of structured graph data through an encoder and decoder. MGAVAE can be used to extract the low-dimensional features of microbes and drugs. The MGAVAE model consists of multiple encoding and decoding layers, which have the same number of layers, and the multiple encoders can improve the learning ability of the model.

The input of MGAVAE is the feature matrix MX or DX and the adjacency matrix MA or DA calculated form the comprehensive microbial or drug similarity matrix MS or DS and the microbe–drug similarity matrix MD . The key point of the variational autoencoder is the application of a two-layer graphical neural network structure for generating low-dimensional representations. The first layer of the neural network is used to compute a low-dimensional feature matrix X :


(12) $$X=\mathrm{GCN}\left(\mathrm{X,}\tilde{A}\right)=\mathrm{LeakyReLU}\left({\tilde{A}\mathrm{XW}}_{0}\right)$$

(13) $$\tilde{A}=d^{-\frac{1}{2}}Ad^{-\frac{1}{2}}$$

where $\tilde{A}$ is the symmetric normalized adjacency matrix, LeakyReLU is the activation function, and $W_{0}$ is the weight parameter of this layer of graph neural net.

In the second layer of the graph neural network, the mean and variance vectors of the feature matrix are computed using the weight parameter $W_{1}$ :


(14) $$\mu ={\mathrm{GCN}}_{\mu }\left(X,A\right)=AXW_{1}$$

(15) $$\log\sigma^{2}=GCN_{\sigma }\left(X,A\right)=AXW_{1}$$

where the mean and variance share the same class of weight coefficients. A reparameterization method is used to compute the obtained low-dimensional features:


(16) Z=μ+σ⊙ε

where ε∈Norm(0,1) represents the standard normal distribution. Here, the decoder is implemented in terms of the matrix inner product, and hence the adjacency matrix is reconstructed as follows:


(17) $$P\left(A|Z\right)=\sigma \left(Z\cdot Z^{T}\right)$$

Finally, the loss function contains two types of errors. The first type is the reconstruction error, which measures the direct similarity of the input and output adjacency matrices. The second type of error is to make the initial label q and the predicted label p as close as possible. The mathematical expression for the loss function is as follows:


(18) $$L=E_{q\left(Z\mathrm{|,X|,A}\right)}\left[\log p\left(A\mathrm{|Z}\right)\right]-KL\left(q\left(Z\mathrm{|,}X\mathrm{|,}A\right)∥p\left(Z\right)\right)$$

where KL(.) represents the Kullback–Leibler divergence between two probability distributions. Finally, the generated low-dimensional features are integrated to obtain the final input for the prediction model.

### 3.3 Graph convolutional neural networks

In this article, modified graph convolution (MGC) is embedded into a variational autoencoder for computing low-dimensional features. Modified graph convolution is modified on the original graph convolution neural network (GCN) to correct the model complexity and redundant computation. The calculation is simplified, and the efficiency is improved by reducing the collapsed weight matrices and non-linearities between successive layers, where the convolution kernel is modified as follows:


(19) $$Y_{MGC}=Softmax\left(S\ldots SSXM^{\left(1\right)}\ldots M^{\left(K\right)}\right)=Softmax\left(S^{K}XM\right)$$

where S is the normalized adjacency matrix, X is the feature matrix, M is the weight matrix, and Softmax represents the normalized exponential function.

### 3.4 Graph attention networks

The graph attention network learns the representation of nodes on the graph through the attention mechanism, assigning different learning weights to different neighboring nodes so that the correlation between node features is better integrated into the model and better prediction performance is obtained (Bian et al., 2021). It uses the graph as an input, including the structural information of the graph and the graph node features. For a two-part graph consisting of microbe and drug associations, the node features are constructed as $Z=\left[\begin{array}{c} Z_{m}\\ Z_{d} \end{array}\right]$ , and the adjacency matrix of the graph is constructed as follows:


(20) $$M=\left[\begin{array}{cc} 0 & A\\ A^{T} & 0 \end{array}\right]$$

where A is the association matrix of microbes with drugs.

A linear transformation of the input features to enhance their expressiveness is defined as follows:


(21) $$Z^{\prime }=Z\cdot W_{Z}$$

where $W_{Z}$ is a learnable weight matrix.

The core idea of graph convolutional networks is to aggregate domain information to update node features. Considering that the importance of different nodes is not the same, a self-attention mechanism is used on the nodes to compute the non-normalized attention coefficient $e_{ij}$ using the current node i and its first-order neighboring node j , defined as follows:


(22) $$e_{ij}=LeakyReLU\left(\left(Z_{i}^{\prime }∥Z_{j}^{\prime }\right)\right)\cdot W_{e}$$

where ∥ represents the concatenation operation, LeakyReLU is the activation function, and $W_{e}$ is the learnable matrix.

The attention coefficient after using the softmax normalized exponential function is calculated as follows:


(23) $$a_{ij}=\frac{\exp\left(e_{ij}\right)}{\underset{k\epsilon N_{i}}{\sum }\exp\left(e_{ik}\right)}$$

where $N_{i}$ represents the node i of the first-order neighbor node.

The first-order neighborhood features are updated by aggregating the l aggregation of first-order neighborhood features with attention coefficients in the layer, updating the l+1 node features of the layer, which are defined as follows:


(24) $$Z_{i}^{l+1}=LeakyReLU\left(\underset{k\epsilon N_{i}}{\sum }a_{ik}^{l}{Z^{\prime }}_{ik}^{l}\right)$$

Utilizing multi-head attention to expand model capabilities and stabilize the learning process. In this process, the initial node features have a dimension of $Z_{m}$ , first, the initial nodes are replicated to obtain M feature matrices of size $\left(N,Z_{m}\right)$ , where N represents the number of nodes. Each replicated feature matrix is then processed with different weight matrices $W_{Z}^{h}$ to compute the outputs of M attention heads. The dimension of each attention head’s output is the same as the initial node feature dimension. Subsequently, the outputs are concatenated to obtain l+1 layer output feature of dimension $\left(N,M\times Z_{m}\right)$ . The output feature of each node i can be calculated as follows:


(25) $$Z_{i}^{l+1}={∥}_{h=1}^{M}LeakyReLU(\underset{k\epsilon N_{i}}{\sum }a_{ik}^{l}{Z^{\prime }}_{ik}^{l}\cdot W_{Z}^{h})$$

where $W_{Z}^{h}$ represents the weight matrices; each attention head has its own weight matrix. During model training, weight matrices are randomly initialized and continuously adjusted through optimization algorithms to minimize the model’s loss function and obtain suitable weight matrices.

### 3.5 Random forest

The random forest algorithm is a well-known integrated learning method. The core idea is to build a forest in a random way, which consists of many decision trees, and the decision trees are used as the base classifiers to form a large multi-classifier. When test data were inputted into the model, the output categories of multiple decision trees were voted to get the final prediction. A decision tree is actually a process of node split, which starts from the root node and continuously splits downward. Knowing that the dataset can no longer be split, the decision tree stops growing.

The core idea is to select n samples from the training set as a training subset and then generate a decision tree, which is a base classifier, the above process is repeated a total of n times, generating n decision trees to form the final random forest. Each base classifier can participate in decision-making and for classification. The final result of the model is decided by the voting of each base classifier, and the class label with the largest number of votes for the classification result is selected.

## 4 Results

### 4.1 Evaluation indicators

In this article, five-fold cross-validation and ten-fold cross-validation will be used to evaluate the prediction performance of the computational model. In the case of the five-fold cross-validation, the specific steps are as follows: first, all the microbe–drug association pairs were divided into five subsets. Each subset was saved individually as a test set; in turn, the remaining four subsets were used as a training set instead of selecting a microbe–drug association pair from them as a test sample. After cross-validation of the model, the receiver operating characteristic (ROC) curve is usually plotted, and the AUC (area under the ROC curve) is calculated to visually assess the predictive performance of the computational model. For a comprehensive evaluation of this computational model, we also evaluate its performance using accuracy (Acc), precision (Pre), recall (R), F1 score, and area under the accuracy-recall curve (AUPR).

The formula for Acc, Pre, R, and F1 score is as follows:


(26) $$\mathrm{Acc}=\frac{\mathrm{TP}+\mathrm{TN}}{\mathrm{TP}+\mathrm{TN}+\mathrm{FP}+\mathrm{FN}}$$

(27) $$\mathrm{Pre}=\frac{\mathrm{TP}}{\mathrm{TP}+\mathrm{FP}}$$

(28) $$R=\frac{\mathrm{TP}}{\mathrm{TP}+\mathrm{FN}}$$

(29) $$F1\;\mathrm{score}=\frac{2\times \mathrm{Pre}\times R}{\mathrm{Pre}+R}$$

where TN is true negative, indicating the number of microbe–drug non-associations correctly identified by the model in the negative samples. TP is true positive, indicating the number of microbe–drug associations correctly identified by the model in the positive samples. FN is false positive, indicating the number of microbe–drug non-associations incorrectly predicted as microbe–drug non-associations by the model in the positive samples. FP is false positive, indicating the number of microbe–drug associations incorrectly predicted as microbe–drug associations by the model in the negative samples and the number of microbe–drug associations in the negative sample.

**Figure 2:**
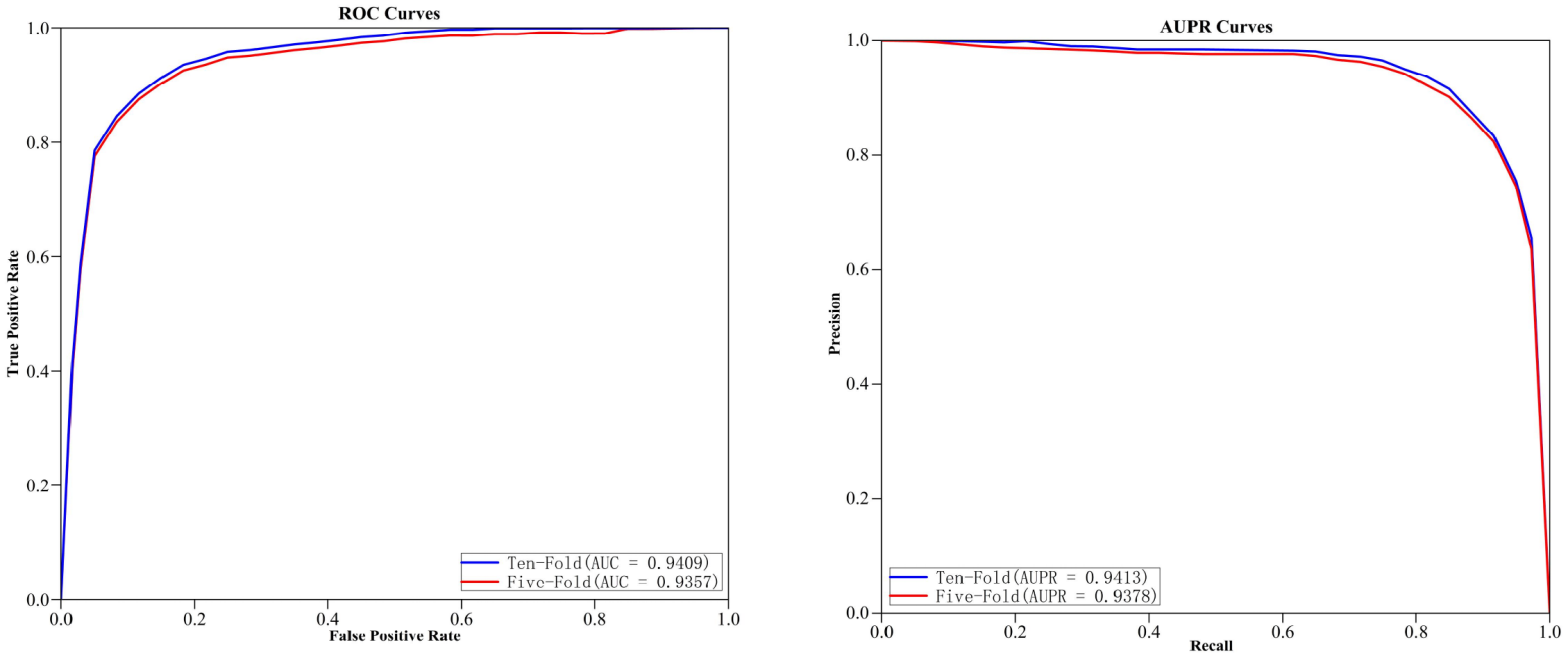
Five-fold and ten-fold cross-validation results of MGAVAEMDA.

The curves of ROC and AUPR for MGAVAEMDA obtained after five-fold and ten-fold cross-validation are shown in Figure 2.

As can be seen from the results of the figure, the AUC and AUPR values of the MGAVAEMDA model on ten-fold cross-validation are 0.52 and 0.35% points, which is higher than the five-fold cross-validation. These data indicate that the MGAVAEMDA model utilizes more training data using ten-fold cross-validation. It can more accurately evaluate the performance of the MGAVAEMDA model in the prediction of microbe–drug association.

### 4.2 Influence of parameter selection

The analysis of the parameters can quantitatively assess the stability of the model (Li et al., 2020). In order to obtain more accurate prediction results, the influence of different parameters on the prediction results was analyzed through experiments. The parameters are divided into three parts: the parameters in MGAVAE, the parameters in the random forest classifier, the parameters of the binarization threshold, and the parameters of the smooth window.

#### 4.2.1 Parameter selection in MGAVAE

##### 4.2.1.1 Hidden layer dimension

**Figure 3:**
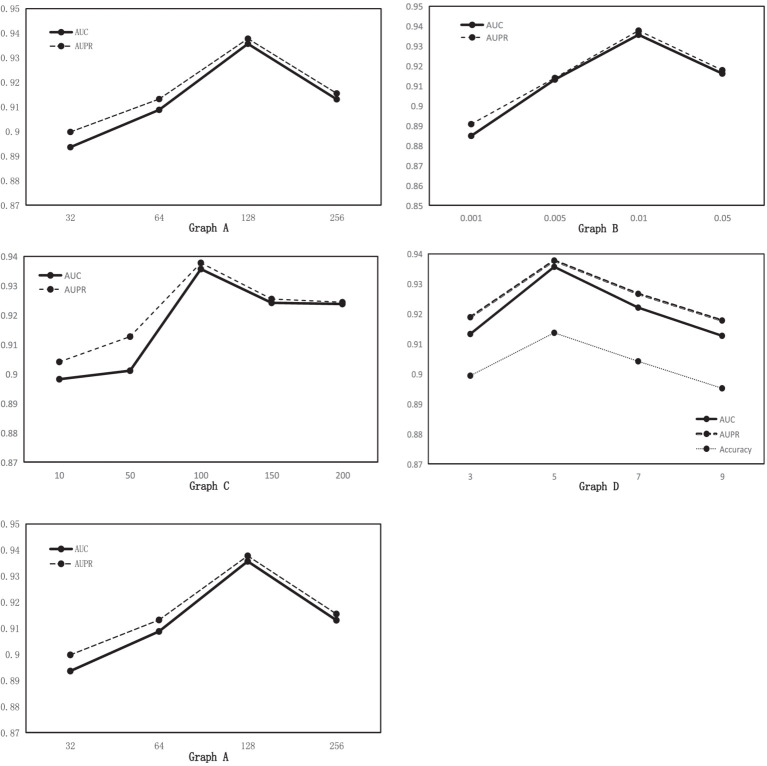
Graph A: AUC and AUPR values for different hidden layer dimensions. Graph B: AUC and AUPR values for different learning rates. Graph C: AUC and AUPR values for different n_estimators. Graph D: AUC, AUPR, and Acc values for different windows.

The fixed learning rate k was set at 0.01 according to the literature to analyze the effect of hidden layer dimension d on the performance of MGAVAEMDA. For each $d\varepsilon \left\{32,\;64,\;128,\;256\right\}$ , five-fold cross-validation, the corresponding AUC and AUPR values are obtained, as shown in Figure 3 Graph A. According to Figure 3, the higher the hidden layer dimension, the smaller the error, but it will increase the complexity of the model and may also be overfitting. From the data, when the dimensionality increases from 32 to 128, the performance of MGAVAEMDA increases with it. When the dimensionality is 256, the values of AUC and AUPR are 1.17 and 1.07% points lower than the dimensionality of 128, respectively. The values of AUC and AUPR of the model are maximum when the dimension is 128. Therefore, setting the hidden layer dimension *d* to 128 ensures the model prediction performance and saves time cost.

##### 4.2.1.2 Learning rate

Fix the hidden layer dimension d to 128 and change the learning rate to a common value. For each $k\varepsilon \left\{0.001,\;0.005,\;0.01,\;0.05\right\}$ , five-fold cross-validation, the corresponding AUC and AUPR values are obtained, as shown in Figure 3, Graph B. According to Figure 3, the values of AUC and AUPR are highest when the learning rate k is 0.01.

#### 4.2.2 Parameter selection in the classifier

##### 4.2.2.1 n_estimators

n_estimators is the number of weak learner. Generally speaking, if n_estimators is too small, it is easy to underfit. If n_estimators is too large, it is easy to overfit, so we usually choose a moderate value. For random forest, increasing the number of “sub-models” (n_estimators) can significantly reduce the variance of the overall model and will not have any effect on the bias or variance of the sub-models. The accuracy of the model increases with the increase in the number of sub-models, and there is an upper limit to the increase in accuracy because the reduction is the second term of the overall model variance formula. In this article, in order to obtain the optimal n_estimators, for every $n\_estimators\varepsilon \left\{10,\;50,\;100,\;150,\;200\right\}$ , five-fold cross-validation, the corresponding AUC and AUPR values are obtained, as shown in Figure 3 Graph C. According to Figure 3, when n_estimators increase from 10 to 100, the performance of MGAVAEMDA increases with it from 100 to 200. The performance of MGAVAEMDA decreases with it, and when the value of n_estimators is 100, the accuracy arrives at the highest level and the overall variance is the smallest. Therefore, n_estimators is set as 100.

#### 4.2.3 Parameter selection in the smoothing window

##### 4.2.3.1 Smooth window size n

Using a smoothing window to reduce the feature noise in the similarity matrix may lead to the loss of useful information in the similarity network. Adjusting the size of the smoothing window is the key. Too large a window may lead to loss of detailed information, while too small a window may not be effective for noise reduction. Therefore, several experiments are needed in the smoothing process to find the most suitable window size. For each $n\in \left\{3,\;5,\;7,\;9\right\}$ , five-fold cross-validation, Figure 3 Graph D shows that when the window size is 5, AUC, AUPR, and Acc are the highest, so smooth window is set as 5.

#### 4.2.4 Parameter selection in binarization

##### 4.2.4.1 Thresholds (*m*th) and (*d*th)

**Figure 4:**
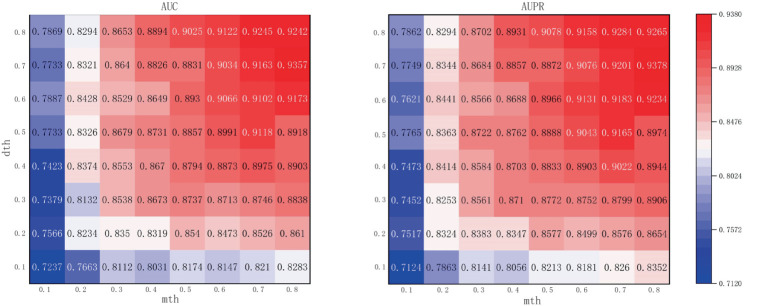
Performance with different combinations of the two hyperparameters.

The *m*th and *d*th are the threshold points for binarization of microbe-integrated similarity networks and drug-integrated similarity networks, respectively. Higher thresholds can effectively reduce the noise in the similarity network but also eliminate the useful information in the similarity network. As shown in Figure 4, the performance of the model gradually improves with the increase of *m*th and *d*th, and the AUC and AUPR reach the maximum when *m*th and *d*th reach 0.8 and 0.7, respectively. In order to ensure that there is more useful information in the similar network and to achieve the best performance of the model, this article considers that it is most appropriate to set *m*th and *d*th to 0.8 and 0.7, respectively.

### 4.3 Comparison of different datasets

**Figure 5:**
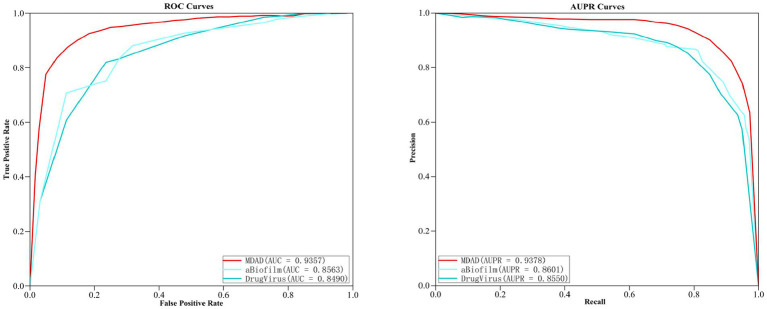
Comparison of prediction results on different datasets.

In order to further verify the prediction ability of the MGAVAEMDA model, this article conducted extension experiments on both aBiofilm and DrugVirus, two microbe–drug databases. After the five-fold cross-validation, the ROC curves and AUPR curves of the three datasets were obtained, which are shown in the following figure. As shown in Figure 5, MGAVAEMDA achieved AUC values of 0.9357, 0.8563, and 0.8490 and AUPR values of 0.9378, 0.8601, and 0.8550 on the MDAD, aBiofilm, and DrugVirus datasets, respectively. The experimental results showed that MGAVAEMDA achieved AUC values and AUPR values that achieved more than 0.8400 and 0.8500 prediction results on different datasets. The results indicate that the model is robust and can be applied to different scales of data.

### 4.4 Comparison with existing methods

**Figure 6:**
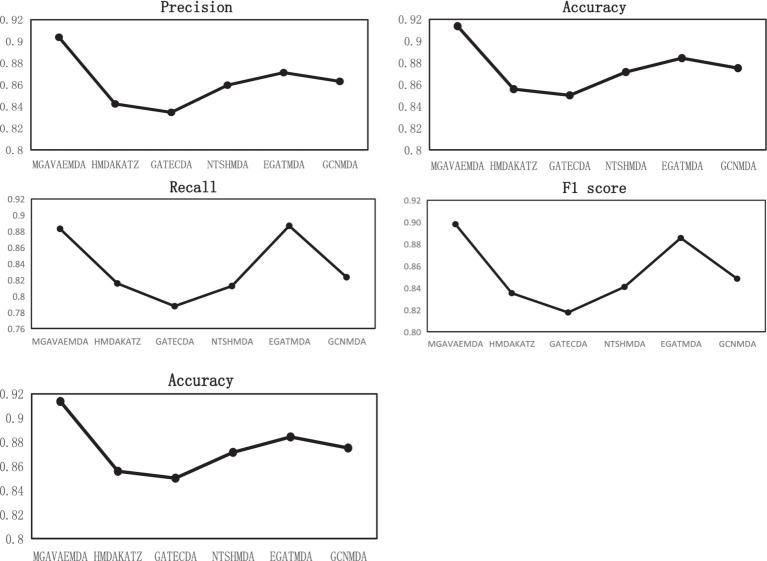
PR curve, ROC curve, Pre, ACC, R, and F1 score curves comparison of the proposed model and existing models.

Under the same dataset conditions, the performance of MGAVAEMDA is compared with five advanced models: HMDAKATZ, GATECDA (Deng et al., 2022), NTSHMDA (Luo and Long, 2020), EGATMDA, and GCNMDA using AUC, AUPR, Acc, Pre, R, and F1 score as the evaluation indicators, and the parameters involved are selected to be the optimal ones recommended in the respective models. The ROC curves and AUPR curves after the five-fold cross-validation are shown in Figure 6.

MGAVAEMDA is superior to the other five groups of comparison experiments, as shown in Figure 6. The AUC values are higher by 4.6, 5.54, 3, 1.76, and 2.92%, and the AUPR values are higher by 4.42, 5.33, 2.66, 1.47, and 2.54%, respectively. For the other four evaluation indicators, MGAVAEMDA is also better than the other five comparison experiments.

Thus, the MGAVAEMDA model has better predictive performance.

### 4.5 Ablation experiments

Ablation experiments are performed by progressively removing modules from the model to assess how much these modules contribute to the overall performance.

(1) To verify the effect of the introduced self-attention mechanism, reconstruction loss function, and KL divergence loss function on the three times improvement of the prediction accuracy of the model MGAVAEMDA, this article conducts four sets of comparative experiments: (1) Group 1: introducing the KL divergence loss function; (2) Group 2: introducing the reconstruction loss function; (3) Group 3: introducing the attention mechanism; (4) Group 4: blank experiment.

**Figure 7:**
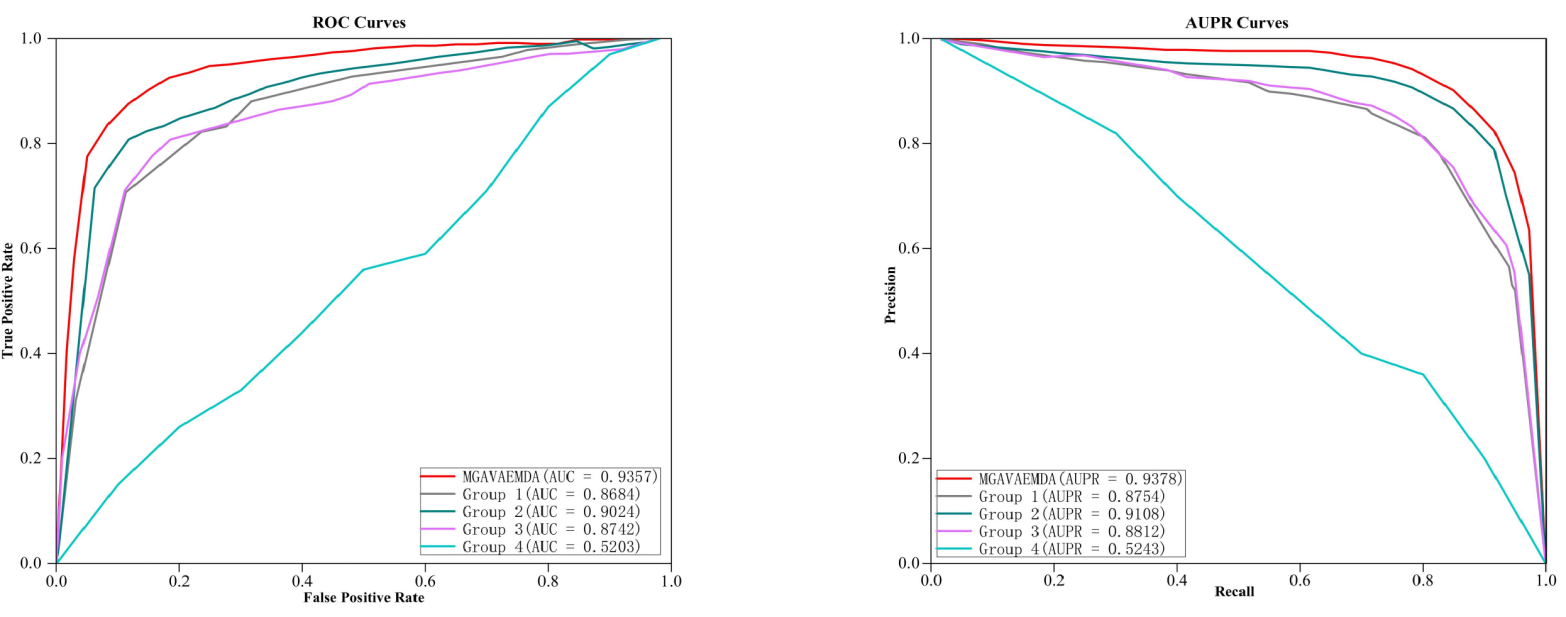
Comparison of ablation experimental results.

After five-fold cross-validation, the ROC curves and AUPR curves of the four group comparison experiments were obtained, which are shown in Figure 7. As can be seen in Figure 7, both the AUC and AUPR values obtained from the MGAVAEMDA model are better than the results from four groups of comparison experiments. Among them, the AUC value of the first group is 34.81% higher than that of the fourth group. The AUPR value is 35.11%, which is higher than the results of the fourth group. It indicates that the addition of the KL divergence loss function reduces the error and improves the optimization ability of the model. The AUC value of the second group is 38.21% higher than that of the fourth group. The AUPR value is 38.65% higher than that of the fourth group, which indicates that the addition of the reconstruction loss function also reduces the error and improves the optimization ability of the model. The AUC value of the third group is 35.39%, higher than that of the fourth group. The AUPR value is 35.69 higher than that of the fourth group, indicating that the addition of the self-attention mechanism reduces the dependence on other information and significantly helps in the fusion of microbe medicines. The AUC value of the MGAVAEMDA is higher than that of groups 1, 2, and 3 by 6.73, 3.33, and 6.15%, and the AUPR values were 6.24, 2.70, and 5.66%, higher than those of the first, second, and third groups, respectively. Those results indicate that the combination of the KL divergence loss function. The reconstruction loss function and the self-attention mechanism can obtain more useful information, which can improve the prediction accuracy of the model. All the groups have shown that the introduction of the KL divergence loss function, reconstruction loss function, and attention mechanism is crucial for the improvement of the prediction accuracy of MGAVAEMDA.

(2) To verify the effect of the introduced modified graph convolutional neural network on improving the computational efficiency of the model MGAVAEMDA. This article sets up a comparative experiment with fixed hardware equipment. The running time of the model without modification is 422 s, and the running time of the model after modification is 353 s, which is significantly improved, indicating that the introduction of the modified graph convolutional neural network improves the computational efficiency of the model.

### 4.6 Case studies

To evaluate the predictive effectiveness of the MGAVAEMDA model, two case studies were conducted using ceftazidime and curcumin. After calculating the predicted microbes associated with these two drugs, the top 10 microbes were screened after arranging the obtained association prediction scores in descending order, as shown in Tables 1, 2.

**Table 1:** Top 10 microbes related to ceftazidime.

Ranking	Name of microbe	PMID number
1	*Stenotrophomonas maltophilia*	37615040
2	*Haemophilus influenzae*	6376458
3	*Shigella flexneri*	31519769
4	*Escherichia coli*	37574665
5	*Pseudomonas aeruginosa*	34990760
6	*Bacillus subtilis*	31420587
7	*Mycobacterium tuberculosis*	28875168
8	*Mycobacterium avium*	28922808
9	*Streptococcus pneumoniae* serotype 4	8126192
10	*Proteus vulgaris*	19802966

**Table 2:** Top 10 microbes related to curcumin.

Ranking	Name of microbe	PMID number
1	*Streptococcus mutans*	23778072
2	*Proteus mirabilis*	21808656
3	*Vibrio anguillarum*	31930829
4	*Pseudomonas aeruginosa*	32421995
5	*Haemophilus influenzae*	27538525
6	*Vibrio cholerae*	35140698
7	*Burkholderia cenocepacia*	Unverified
8	*Enterococcus faecalis*	34320428
9	*Burkholderia multivorans*	Unverified
10	*Eikenella corrodens*	Unverified

The prediction data of the above two tables were obtained from the MGAVAEMDA model by searching the PMID database for relevant literature and reports. The data in the table show that among the top 10 microbes predicted in the MGAVAEMDA model with ceftazidime and curcumin, 10 and 7 each have been confirmed in the literature. Among them, curcumin-mediated EDTA blue light PDI has a strong inhibitory effect on *Streptococcus mutans* in planktonic culture. It is expected to be a promising technique for disinfection of oral tissues due to its unclear targeting mechanism (Nima et al., 2021). Curcumin has an inhibitory effect on urease activity in *Proteus mirabilis* (Prywer and Torzewska, 2012), and the addition of curcumin increases the induction time and decreases the growth efficiency of guano stones as compared to the absence of curcumin. Curcumin has been used in the case of *Proteus mirabilis*-induced growth of guano crystals in association with urinary stone formation, which has been shown to have great potential for further research. The case study further validates that the MGAVAEMDA model has a good performance in identifying microbe-associated drugs and has a certain application value.

## 5 Conclusion

In this study, the MGAVAEMDA model was set up by using the variational autoencoder, modified graph convolutional neural network, and graph attention network to realize the microbe–drug association prediction through two-stage fusion.

The case study indicates that the MGAVAEMDA model can overcome the shortcomings of other models, such as long training times and low prediction accuracy. Moreover, the MGAVAEMDA model has better prediction performance.

The predicted performance of the model will be improved after more biological datasets are integrated in the future, so this model’s practical application will increase.

## Data availability statement

The raw data supporting the conclusions of this article will be made available by the authors, without undue reservation.

## Author contributions

BW: Writing – review & editing. FJM: Writing – editing. XXD: Data curation, Writing – review & editing. GDZ: Formal analysis, Writing – review & editing. JYL: Investigation, Writing – review & editing.
